# Comparison of the Effects of GMCSF-Containing and Traditional Culture Media on Embryo Development and Pregnancy Success Rates

**DOI:** 10.1055/s-0042-1759630

**Published:** 2022-12-29

**Authors:** Fatih Adanacıoglu, Çağlar Çetin, Gozde Tokat, Durdane Adanacıoglu, Ayse Filiz Gokmen Karasu, Mehmet Turan Çetin

**Affiliations:** 1 Turan Cetin Private IVF Center, Adana, Turkey; 2 Department of Obstetrics and Gynecology, Bezmialem Vakif University School of Medicine, Istanbul, Turkey; 3 Department of Embryology, Turan Cetin Private IVF Center, Adana, Turkey; 4 Adana State Hospital, Adana, Turkey

**Keywords:** in vitro fertilization, embryo culture, embryo transfer, granulocyte macrophage colony-stimulating factor

## Abstract

**Objective** The use of granulocyte macrophage colony-stimulating factor (GM-CSF)-containing medium, which is a commercial medium that is used for cultivation of embryos in in vitro fertilization (IVF) treatments, has been suggested to increase the efficiency of this procedure in patients with previous multiple unsuccessful attempts. In this retrospective study, we analyzed GM-CSF-containing embryo culture media compared with traditional culture media in terms of development of embryos, pregnancy, and ongoing pregnancy success and live birth rates.

**Methods** This is a prospective case control study conducted in a single center. A total of 131 unexplained infertility patients were included in the study. A cohort of 69 patients whose embryos were cultured in GM-CSF-containing medium and a control group of 62 age-matched patients whose embryos were cultured in conventional Sage One Step medium were included in the study. The major study outcomes were achievement of pregnancy and ongoing pregnancy rate at 12 weeks of gestation.

**Results** The pregnancy and ongoing pregnancy rates of the patients whose embryos were cultured in GM-CSF-containing medium were 39.13% and 36.23%, respectively. These were higher than the rates of the control group, which were 30.65% and 29.03%, respectively, although this difference was not statistically significant. In addition, the 5 ^th^ -day embryo transfer percentage in the GM-CSF group was higher than in the control group (34.78% versus 27.4%).

**Conclusion** The main findings of our study were that there was no difference between the GM-CSF-enhanced medium and the control group in terms of our major study outcomes. However, blastomere inequality rate and embryo fragmentation rates were lower in the GM-CSF group.

## Introduction

Ongoing research in assisted reproductive technology, especially in vitro fertilization (IVF) methods, has increased the efficiency of treatment. The important problems associated with IVF treatment include implantation failures and miscarriages. The arrest of embryonic development results in miscarriage in some IVF-assisted pregnancies. Repeated implantation failure, which occurs when embryos fail to implant following several IVF treatment cycles, can be caused by reduced sensitivity of the endometrium, genetic factors, or stress. The success of implantation in the IVF process depends on three main parameters: a healthy embryo, a receptive endometrium, and a strong communication between these two to facilitate the subsequent events necessary for pregnancy. 1 The communication between the embryo and the endometrium is made possible by the interaction of cytokines, which are necessary for the regulation of normal embryonic development, enhancing implantation efficiency and normal development of the fetus and the placenta. 2 3 4 5

There are some differences between the in vitro culture conditions utilized during the IVF process and the in vivo conditions. 6 7 8 9 10 The lack of necessary ingredients and factors during in vitro culture may cause recurrent implantation failure and other clinical pathologies. 7 11 12 Since the culture conditions and the ingredients of the in vitro culture medium are important to ensure the success of the IVF treatment, there is ongoing research to find the optimal conditions for the in vitro culture and its effect on the embryo development and the health of the baby. Addition of the cytokines that are responsible for the embryo-endometrium communication to the culture medium during IVF has been shown to be important in normal blastocyst development, embryo implantation, and development of the fetus and placenta. 13 14 15 16

Numerous cytokines and growth factors which are secreted by the endometrium have exhibited in stimulating normal embryo development. Among the above-mentioned factors, there has been a focus on granulocyte-macrophage colony-stimulating factor (GM-CSF), also known as CSF (colony stimulating factor). Granulocyte-macrophage colony-stimulating factor is expressed in the epithelial cells lining the oviduct and uterus 17 and enhances embryo implantation and development of a normal embryo. 18 19 20 21 22 The embryos, which invade placental trophoblast cells and the abundant populations of leukocytes controlling maternal immune tolerance, are affected by GM-CSF adjustment. Granulocyte-macrophage colony-stimulating factor deficiency in pregnancy negatively alters fetal and placental development. Additionally, GM-CSF regulation is also important in growth after birth; thus, this cytokine is a central maternal determinant of pregnancy outcome with clinical relevance in human fertility. Preclinical studies show that GM-CSF has positive effects on development and implantation efficiency in mouse and human embryos. 20 21 22

During early pregnancy, GM-CSF in blood serum increases dramatically, and the levels decrease if pregnancy is terminated. 23 Reduced GM-CSF levels have been associated with miscarriage. ^4^ Research on the use of GM-CSF in the IVF process has shown promising results. 16 The addition of recombinant GM-CSF to the culture medium has been shown to increase the success rate of IVF. 24 25 26

## Methods

The institutional review board (71/10/2016) approved the present study. Informed verbal and written consent were obtained from all participating couples. A total of 131 unexplained infertility patients who received IVF treatment were chosen to participate in the study or the control group. Embryos of 69 of these patients were treated with GM-CSF-containing medium, whereas 62 were treated with conventional medium.

All embryos in the control group were incubated with single-step culture medium (without GM-CSF). For the control group selection, matching was done considering female age, body mass index, number of M-II oocyte retrieved, and number of embryos transferred. All patients were treated between 2016 and 2018. The sperm parameters of all patients who participated in this study were evaluated according to the World Health Organization standards, and morphology was assessed by the Kruger criteria. The total sperm counts of all patients were over 15 million. The mean forward mobility was over 32%. The sperm morphologies were between 3 and 7%, according to the Kruger criteria. All patients had normal hysterosalpingography (HSG). The exclusion criteria were as follows: patients carrying genetic anomaly risk; those who had mature but unfertilized oocytes; those who had immature or low-quality oocytes; and patients with uterine cervical insufficiency, thrombophilia, and sperm morphology lower than 3%, according to the Kruger strict criteria.

After oocyte pick up, the oocytes were either transferred to a GM-CSF-containing medium or Sage One Step medium (CooperSurgical, Denmark). In the study group, the oocytes that were collected at day 0 were transferred to GM-CSF-containing medium drops with 35 µL volume. Four hours after oocyte pick-up, denudation and intracytoplasmic sperm injection was performed. On day 1 after fertilization control, the zygotes were transferred to fresh GM-CSF-containing medium drops (Embryogen, Origio, Denmark). After this transfer, their development was monitored daily. In the control group, the oocytes that were collected on day 0 were placed in 35 µL Sage One Step droplets that were coated with paraffin oil. The oocytes were denuded after 4 hours, and an intracytoplasmic sperm injection was applied. After checking for fertilization on day 1, the zygotes were transferred to fresh Sage One Step drops, and their development was monitored daily. The day of embryo transfer was determined according to the development of embryos. The maximum number of transferred embryos was two per patient. Embryos were assessed morphologically at cleavage stage (day ⅔) on the basis of number and symmetry of blastomeres, degree of fragmentation, and presence of multinucleated cells according to the British Fertility Society and Association of Clinical Embryologists guidelines, published by Cutting et al. (2008). 27

After oocyte pick-up, 90 mg progesterone gel was applied vaginally the next day. On the second day posttransfer, 2 mg of estradiol was applied transdermally by using a 6.5 cm ^2^ patch. The day of embryo transfer was determined according to the development of the embryos. Intramuscular daily injections of 50 mg of progesterone and peroral application of 4 mg of methyl prednisolone were performed for a 12-day period after embryo transfer. On day 12 of the transfer, β-HCG levels were tested in serum blood samples obtained from the patients.

The GraphPad Prism software was used for the statistical analyses. The difference between groups was analyzed using the Chi-squared test. The test results were presented with 95% confidence interval. A *p* -value < 0.05 was set as statistically significant. All data are presented as average ± standard deviation (SD).

## Results

The effect of culturing embryos in GM-CSF-containing medium was studied in a total of 131 patients who underwent IVF treatment in the center. Sixty-nine of the patients were in the GM-CSF-containing medium group, whereas 62 patients constituted the control group whose embryos were cultured in Sage One Step. The ages of the patients ranged between 23 and 38. The average ages of the control group and GM-CSF-containing medium group were 30.61 ± 3.46 years and 30.73 ± 3.89 years, respectively. The endometrial thickness values of both groups were also found to be comparable ( Table 1 ). Also, the pregnancy outcomes and live birth ratios of the study group are demonstrated in Table 2 .

**Table 1:** The pregnancy outcomes and clinical information of the study group

Protocol	# of Patients	Mean age ± SD	# of transferred embryos	Endometrial thickness
GM-CSF containing medium	69	30.73 ± 3.89	1.91 ± 0.28	9.36 ± 1.03
• Pregnant	27 (39.13%)	30.18 ± 4.12	1.96 ± 0.19	9.48 ± 0.75
• Not pregnant	42 (60.87%)	31.72 ± 5.62	1.91 ± 0.30	9.13 ± 0.99
**No GM-CSF containing medium**	**62**	**30.61 ± 3.46**	**1.89 ± 0.32**	**9.37 ± 1.11**
• Pregnant	19 (30.64%)	30.89 ± 3.90	1.79 ± 0.42	9.74 ± 1.10
• Not pregnant	43 (69.36%)	30.49 ± 3.29	1.86 ± 0.37	9.22 ± 1.52

**Table 2:** The pregnancy outcomes and live birth ratios of the study group

Protocol	Pregnancy	Ongoing pregnancy	Live birth	Twin birth
GM-CSF-containing medium	27 (39.13)	25 (36.23)	25 (36.23)	4 (5.79)
Non-GM-CSF-containing medium	19 (30.64)	18 (29)	18 (29)	2 (1.24)

The culture medium containing GM-CSF is usually recommended for IVF patients with a history of multiple unsuccessful treatment attempts. We analyzed the number of previous unsuccessful attempts for the GM-CSF-containing medium group and found that the average number of previous unsuccessful attempts was 3.03 ± 1.51. The average number of previous unsuccessful IVF attempts for the control group, on the other hand, was 3.00 ± 0.54. The inequality rates of blastomeres in the GMCSF were lower than in the control group. In the GMCSF group it was 10%, and in the control group the inequality rate was 40%. In addition, in the GM-CSF-containing medium group, 80% of the embryos had less than 5% fragmentation, while in the control group 50% of the embryos had less than 5% fragmentation. Among the control group, the ongoing pregnancy rate at 12 weeks was 30.65%, whereas this rate was 39.13% for the GM-CSF-containing medium group. However, the difference between groups was not statistically significant according to the Chi-squared test with a *p* -value of 0.3106 and an odds ratio of 0.6873 and 95% confidence interval of 0.3330 to 1.4188.

## Discussion

**Fig. 1:**
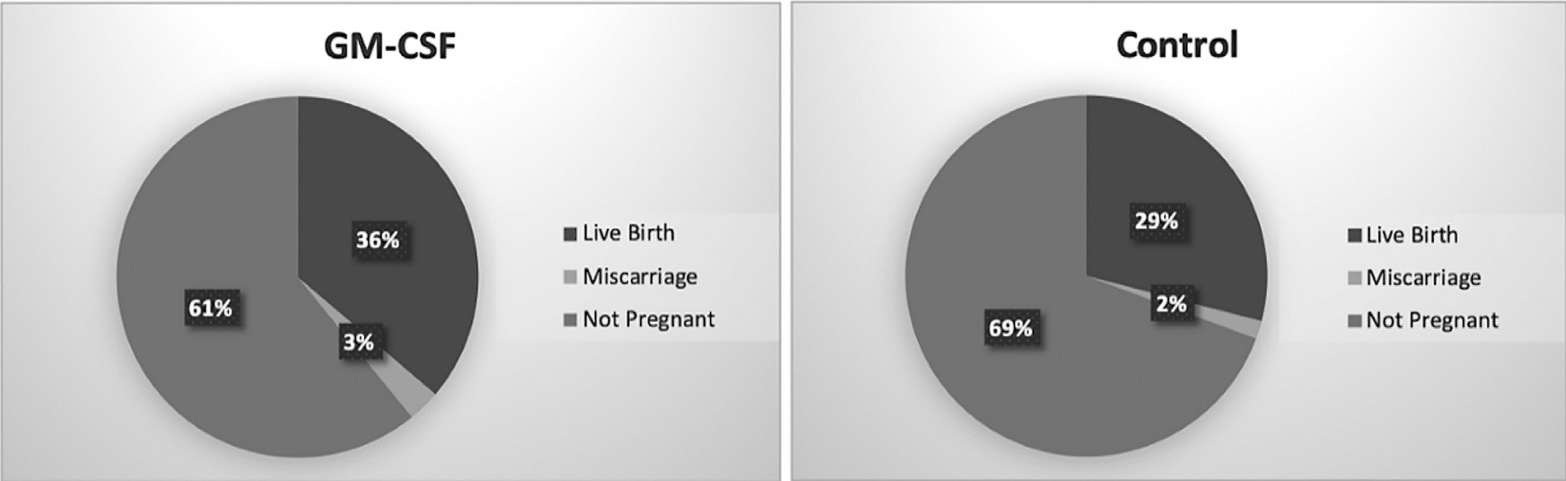
The distribution of single births, miscarriages, and twin births among pregnant women who underwent IVF using either GM-CSF-containing medium or Sage One Step.

The main findings of our study were that there was no difference between the GM-CSF-enhanced medium and the control group in terms of our major study outcomes. However, blastomere inequality rate and embryo fragmentation rates were less in the GM-CSF group. The success of the IVF treatment depends on, among many other factors, the environmental conditions of the embryo before implantation. The presence of growth factors results in increased efficiency of preimplantation embryo development. Among these growth factors, addition of GM-CSF into the culture medium has been shown to have favorable outcomes in preimplantation development and clinical pregnancy rates. Ziebe et al. 26 showed an increase in ongoing implantation rate in women with high incidences of miscarriage, from 17 to 24.5% when GM-CSF-containing medium was used. Among women who were not classified based on previous IVF failures, the ongoing implantation rates at 12 weeks were 23.0% (GM-CSF) and 18.7% (control). The live birth rates were 28.9% (GM-CSF) and 24.1%(control) in the same cohort, respectively. We also observed that the pregnancy rate increased from 29.032 to 39.13% in the GM-CSF-containing medium group compared with control group, but this difference was not statistically significant. This result is in line with the other studies mentioned above ( Fig. 1 ). Also similarly, in our study, the implantation rate was higher in the GM-CSF group too (12 weeks 39.13% vs 30.65%), and the live birth rates were 36.23% (GM-CSF) and 29%(control) in the same cohort, respectively.

**Fig. 2:**
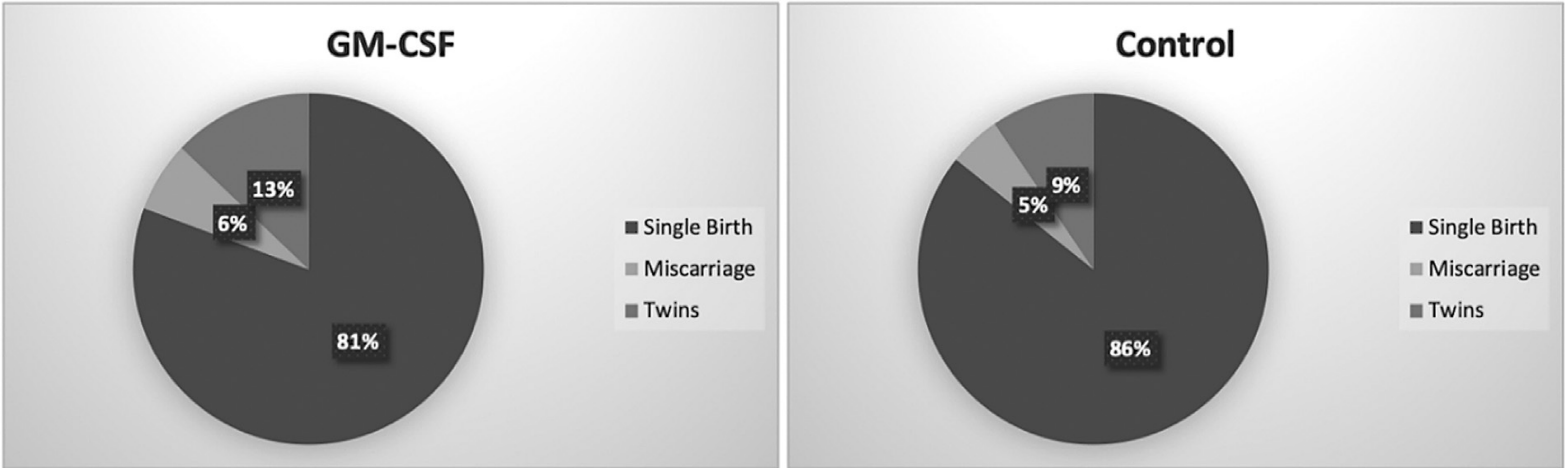
The distribution of pregnant versus non-pregnant patients who underwent IVF using either GM-CSF-containing medium or Sage One Step and their live birth ratios.

Mignini Renzini et al. 24 showed that miscarriage rates were reduced, and live birth rates were increased in women with a history of miscarriage; however, there was no significant difference between the clinical pregnancy rates. The patient population in our study did not experience pregnancy before, there was no history of miscarriage. However, we observed that embryos developed better in GM-CSF medium, which may decrease the miscarriage rates. Sfontouris et al. 21 observed that clinical pregnancy rates (35.3% vs 22.9%), and implantation rates (12 weeks, 17.4% vs 11.4%) were higher in the GM-CSF containing medium group compared with controls, but these differences were not statistically significant. Similarly, in our study, the implantation rate was higher in the GM-CSF group (12 weeks 39.13% vs 30.65%). In another study, Tevkin et al. 28 showed that the frequency of clinical pregnancy increased, and early pregnancy losses decreased in the GM-CSF-containing medium treated group compared with the control group. 27 28 These results suggest that the use of GM-CSF increases the efficiency of IVF treatment in patients with multiple unsuccessful IVF attempts. It was suggested that the transfer of blastocysts to the uterus may lead to higher implantation rates and, thus, reduce the levels of multiple births after IVF treatment. 16 However, Mignini Renzini et al. 24 observed a higher live birth rate (19.5% versus 9.5%) and an increase in twin births in the GM-CSF-treated group, which is similar to our results (5.79%vs 1.24%). The reason for this appears to be a decrease in miscarriage rates; however, there may also be another mechanism involved. More studies will be important to define the mechanism of action through which GM-CSF may affect pregnancy and live birth rates. Singleton and twin births rates in our study are shown in Fig. 2 .

The major limitation of our study is that the study population was small. However, the strengths of our study include prospective design, appropriate follow-up, and reporting of ongoing pregnancy results. Overall, our results are in line with previous observations showing that the addition of GM-CSF into the culture media increases clinical pregnancy by better mimicking the natural in vivo conditions and helping the embryo to better adapt to the in vitro conditions. Our study strengthens the view that clinical pregnancy and ongoing pregnancy rates after IVF treatment in patients with multiple failures may be enhanced by the use of GM-CSF-containing medium in vitro.

## Conclusion

The main findings of our study were that there was no difference between the GM-CSF-enhanced medium and the control group in terms of our major study outcomes. However, blastomere inequality rate and embryo fragmentation rates were lower in the GM-CSF group.
